# Papillary Fibroelastoma as a Cause of Cardioembolic Stroke in Young Adults: A Case Report

**DOI:** 10.7759/cureus.97447

**Published:** 2025-11-21

**Authors:** Fareha Syeda Zahid, Peter Howard, Peter Haworth

**Affiliations:** 1 Internal Medicine, Queen Alexandra Hospital, Portsmouth, GBR; 2 Stroke Medicine, Queen Alexandra Hospital, Portsmouth, GBR; 3 Cardiology, Queen Alexandra Hospital, Portsmouth, GBR

**Keywords:** cardiac papillary fibroelastoma, cardiac tumours, cardioembolism, echo cardiogram, ischaemic strokes, magnetic resonance imaging, primary cardiac tumours, stroke, young adults, young people

## Abstract

Although ischaemic strokes are common worldwide, strokes in young adults are rare. Cardioembolism is a frequent cause of ischaemic strokes, often arising from atrial fibrillation or, less commonly, underlying structural heart defects. Primary cardiac tumours are commonly associated with embolic strokes. Although papillary fibroelastoma (PFE) is a rare cardiac tumor, it is one of the most common primary cardiac tumours that causes embolic strokes. We report the case of a 24-year-old woman who presented with sudden-onset right arm weakness. Subsequent investigations, including echocardiography, revealed a papillary mass consistent with PFE.

## Introduction

Ischaemic strokes are common worldwide. According to NHS England data, there were 111,137 stroke admissions in England in 2023/24 [1]. Whilst most strokes affect older patients, the National Young Stroke Study reports that the incidence in patients under 55 years has doubled over the last 10-20 years, with approximately 20,000 new cases annually in the United Kingdom [2]. 

A cardioembolic stroke is a type of ischemic stroke caused by a blood clot that forms in the heart and travels to the brain, blocking blood flow; structural heart defects are among the possible causes. Papillary fibroelastomas are the second most common benign tumours of the cardiac valves, second only to myxomas. Although histologically benign, their mobility and friable structure can lead to serious complications, including systemic embolization and ischaemic stroke [3]. This report presents a case of a young adult who suffered a stroke secondary to a structural cardiac defect, emphasizing the critical role of echocardiography in evaluating suspected stroke in young patients.

## Case presentation

A 24-year-old woman was referred to our transient ischemic attack (TIA) clinic for assessment. Her most recent event involved sudden-onset right arm weakness and speech difficulty, lasting approximately 15 minutes, followed by complete recovery. Over the preceding year, she had experienced similar transient neurological episodes, including one affecting her left side and causing temporary inability to speak. She also reported recurrent episodes of vertigo, characterized by spinning sensations and imbalance, often accompanied by pins and needles in her face, hands, and arms, as well as an inability to walk during attacks. Additional symptoms included memory lapses, with two or three brief episodes, one of them witnessed, during which she suddenly forgot what she was doing while at work. All episodes were short-lived, typically resolving within 20-30 minutes. She had no significant past medical history. Her only medications were the combined oral contraceptive pill and Aspirin 75 mg once daily, which had been initiated by her general practitioner at the time of referral to the TIA clinic. She had a very healthy lifestyle and was a recreational long-distance runner. She did not smoke cigarettes, take recreational drugs, or drink a significant amount of alcohol.

Clinical observations were within normal limits, and physical examination was unremarkable. An electrocardiogram demonstrated a normal sinus rhythm. Due to her young age, no imaging had been performed prior to the initial appointment. The initial differential diagnosis included complex migraine and focal seizures. An urgent short stroke protocol MRI brain scan was requested to evaluate for any structural abnormalities or evidence of restricted diffusion. The patient was advised to discontinue the combined oral contraceptive pill, continue aspirin monotherapy, and refrain from driving until further assessment.

**Figure 1:**
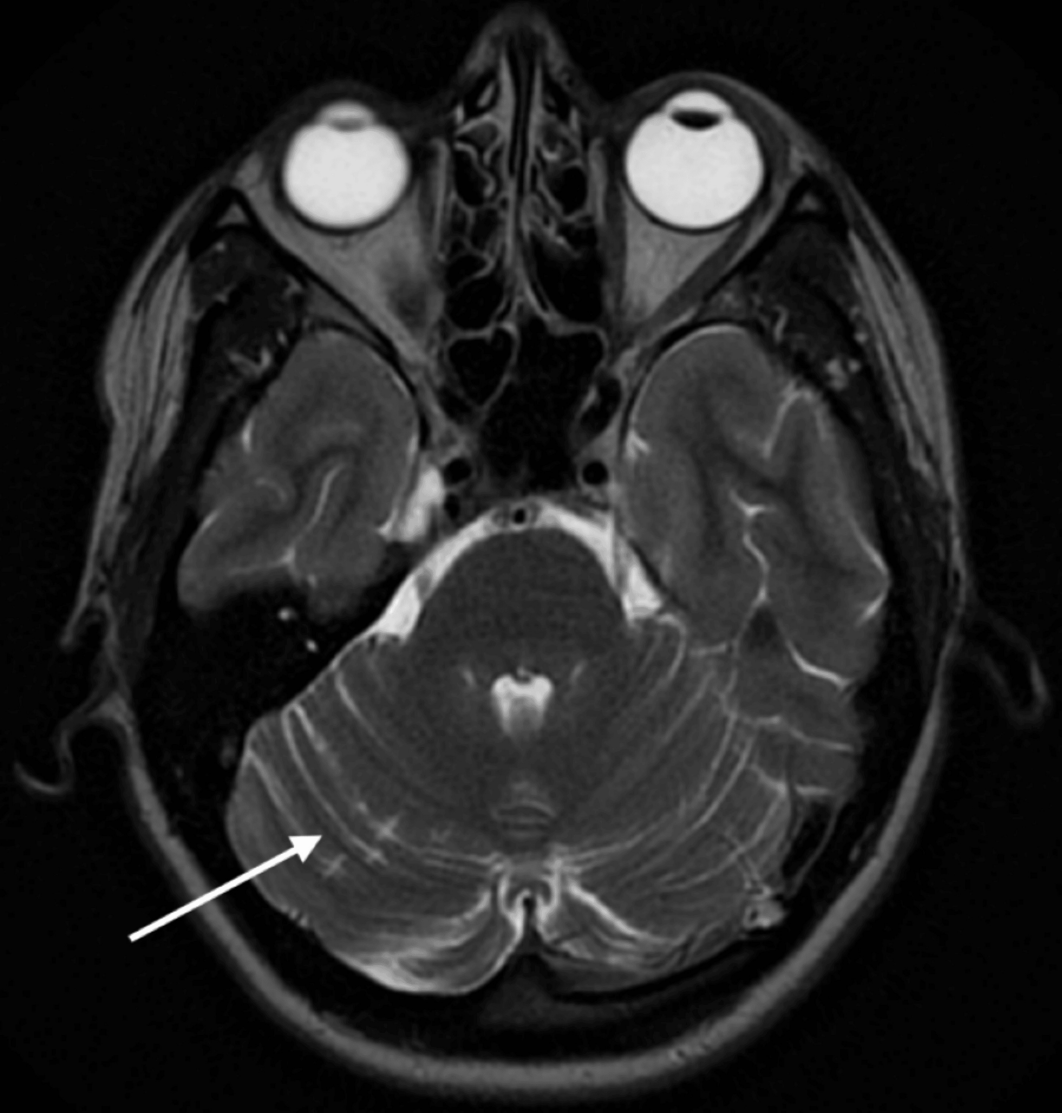
MRI Head (Axial view) demonstrating right sided cerebellar infarcts.

The MRI revealed three old right cerebellar infarcts with no evidence of an acute stroke (Figure 1). The acute presentation was diagnosed as likely complex migraines, with the infarcts considered coincidental findings. Subsequently, the patient also reported headaches accompanied by unilateral visual disturbances. Aspirin was switched to Clopidogrel 75 mg once daily, and regular blood pressure monitoring was advised. Further investigations were requested, including a young stroke blood panel, echocardiography, a 72-hour tape, and a magnetic resonance angiography (MRA) scan of the neck vessels and Circle of Willis.

**Figure 2:**
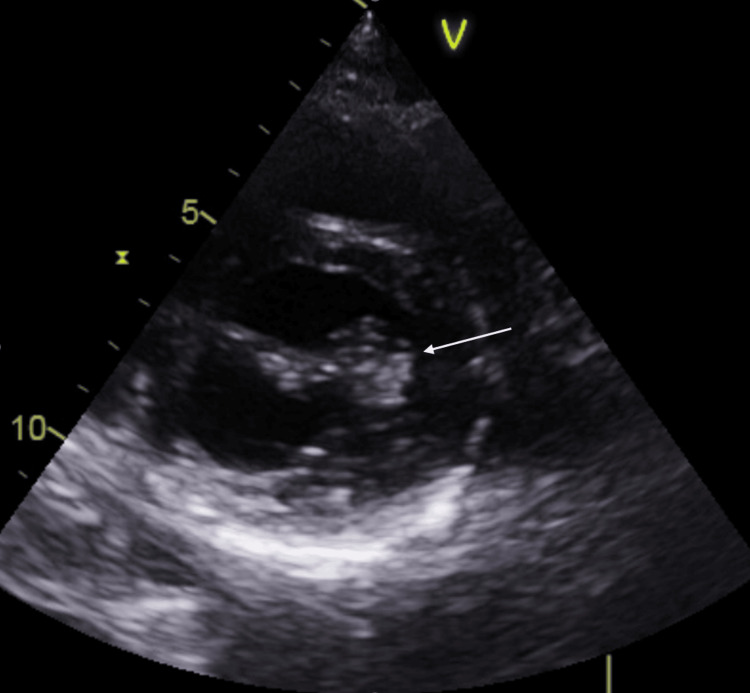
Echocardiogram demonstrating mitral valve mass.

**Figure 3:**
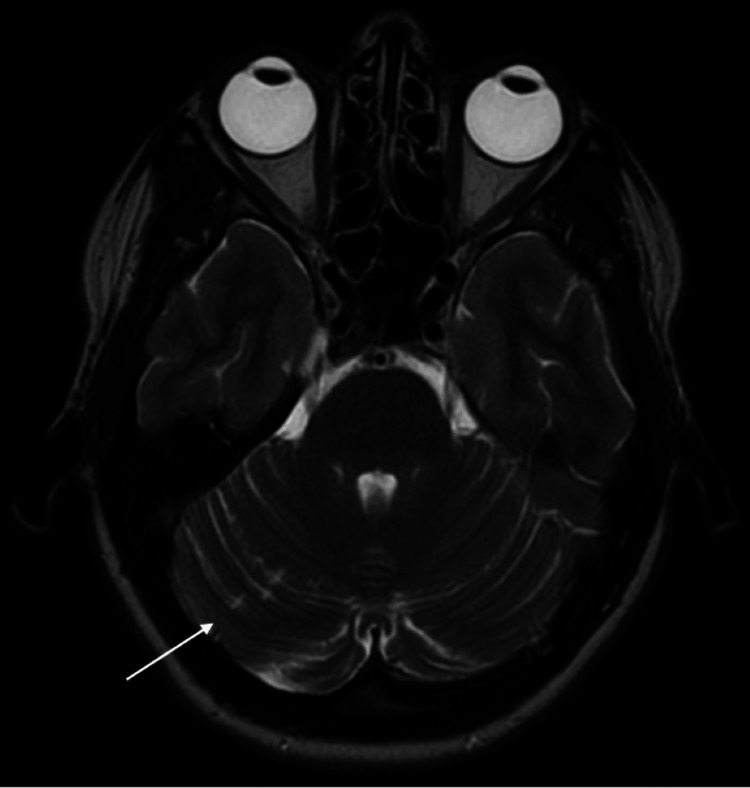
Follow-up MRI Head (axial view) demonstrating multiple small foci in right cerebellar hemisphere, no progression since previous MRI.

All blood tests returned within normal limits, including a test for Fabry’s disease. The MRA scan showed entirely normal arteries. Echocardiography, however, revealed a mobile mass measuring 15 x 16 mm attached to the anterior mitral leaflet chordae as can be seen in Figure 2. The patient was admitted directly from the outpatient echocardiography clinic to a cardiology inpatient unit and subsequently, following a multidisciplinary team discussion, the patient was transferred directly to the local cardiac surgery unit. The patient underwent mitral valve repair and anterolateral commisuoplasty. She had an uneventful six-day inpatient stay. Histopathology confirmed the diagnosis of a papillary fibroelastoma. Follow-up MRI after two months showed similar infarcts consistent with chronic ischaemia (Figure 3). 

## Discussion

Primary tumours of the heart are rare, with a frequency of approximately 0.02% based on data from 22 large autopsy series [4]. Cardiac tumours are classified as benign or malignant, with benign types being much more common. Common benign tumours include myxomas, the most frequent primary cardiac tumour, along with rhabdomyomas, fibromas, lipomas, and papillary fibroelastomas. Most PFEs are diagnosed incidentally; however, their friable, mobile, and highly vascular nature makes them particularly prone to embolization, predisposing patients to ischaemic stroke, transient ischaemic attack, and systemic emboli [5,6].

Transient neurological episodes in young adults are most often due to primary headache disorders. The presence of dual pathologies is unusual but not unexpected, given the incidence of primary headache disorder.

In our patient, the diagnosis would have been missed had she not been referred for an MRI brain scan. Ultimately, a full investigation of the historic infarcts with an echocardiogram was key to revealing a very unusual cardioembolic source, requiring urgent cardiac surgery. Echocardiography played a pivotal role, revealing a very unusual cardioembolic source. This case highlights the importance of comprehensive evaluation in patients with cryptogenic stroke, even when found coincidentally, particularly when routine vascular and haematological investigations are unremarkable.

Current United Kingdom stroke care guidelines recommend that patients with stroke or TIA undergo transthoracic echocardiography (TTE) if identifying a structural cardiac abnormality would alter their management, particularly in cases of unexplained stroke or TIA [7].

PFEs located on the left-sided cardiac valves, particularly the mitral and aortic valves, are reported to have the highest embolic potential [8]. Surgical excision is widely regarded as the treatment of choice, even in asymptomatic patients, when the tumour is large (>1 cm), highly mobile, or associated with previous embolic events [9,10]. In our patient, the combination of young age, lesion size, mobility, and evidence of previous infarcts required surgical intervention. The literature describing excellent postoperative outcomes, including safe valve-sparing resections and low recurrence rates, further supports early excision to prevent recurrent or catastrophic embolic complications [6,11].

Although PFEs are histologically benign, their clinical course can be severe if left untreated. In young patients presenting with recurrent transient ischaemic attacks or cryptogenic strokes, clinicians should maintain a high index of suspicion for rare cardiac tumours. Optimal care in such cases requires a multidisciplinary approach, bringing together neurologists, cardiologists, and cardiothoracic surgeons to ensure timely diagnosis and effective management.

## Conclusions

Early recognition of cardioembolic causes through thorough evaluation, including echocardiography, is critical in patients with cryptogenic or recurrent transient ischaemic attacks. Surgical excision remains the definitive treatment, and a multidisciplinary approach ensures optimal outcomes, minimizing the risk of recurrent embolic complications. This case emphasizes the importance of considering rare cardiac tumours in the differential diagnosis of stroke in younger populations.
